# The discovery of human *Plasmodium* among domestic animals in West Sumba and Fakfak, Indonesia

**DOI:** 10.12688/f1000research.53946.2

**Published:** 2023-02-15

**Authors:** Munirah Munirah, Sitti Wahyuni, Isra Wahid, Firdaus Hamid

**Affiliations:** 1 Doctoral Study Program, Faculty of Medicine, Hasanuddin University, Makassar, South Sulawesi, 90245, Indonesia; 2 Department of Parasitology, Faculty of Medicine, Hasanuddin University, Makassar, South Sulawesi, 90245, Indonesia; 3 Department of Microbiology, Faculty of Medicine, Hasanuddin University, Makassar. Jln. Perintis Kemerdekaan 10 Tamalanrea, Makassar, South Sulawesi, 90245, Indonesia

**Keywords:** Plasmodium falciparum, Plasmodium vivax, malaria, animals, host reservoir, PCR.

## Abstract

**Background**: In Indonesia, malaria incidence is at a high rate despite maximum preventive efforts. Therefore, this study aims to determine the possibility of a *Plasmodium* reservoir among domestic animals in malaria-endemic areas.

**Methods**: Animal blood was collected using EDTA tubes, then smeared and stained with Giemsa for *Plasmodium* microscopic identification. About 10 µl of blood was dropped on to a filter paper to capture *Plasmodium* DNA. Nested PCR was used for parasite molecular detection, while *Plasmodium* species were identified using the sequenced DNA.

**Results**: A total of 208 and 62 animal blood samples were collected from Gaura village, West Sumba and Fakfak village, West Papua, Indonesia respectively. In total, 32 samples from Gaura contained *P. falciparum* or *P. vivax*, while the *Plasmodium* percentage in buffalo, horse, goat, and dogs were 20.7%, 14.3%, 5.8%, 16.7%, respectively. *P. knowlesi* was not found in any of the samples, and no other species were detected in 18 pig blood samples.

**Conclusion:** Human *Plasmodium* existence among domestic animals in Indonesia partly explains the high prevalence and persistence of malaria in some endemic areas due to a reservoir host presence. Therefore, future studies need to ascertain the cause.

## Introduction

Indonesia has made great strides toward becoming a malaria-free region through the Malaria Control Programme. Many efforts have been carried out to eradicate this disease, starting from early detection, treatment and eradication of mosquito vectors, but until now the disease has not been eliminated. In addition, parasite resistance to antimalarial drugs, mosquito resistance to insecticides, and inadequate health system performance. Another factor may make elimination difficult in this region is the presence of a host reservoir.

*Plasmodium falciparum* and *P. vivax* commonly infect humans with high morbidity and mortality in Indonesia. Before molecular diagnostics development, only humans were assumed to be the primary host for *Plasmodium falciparum, P. vivax, P. malariae,* and *P. ovale.* However, studies in the last two decades on *Plasmodium* reported that the parasites originated from animals *.* Further stating that *P. falciparum* originated in the gorilla ( Liu et al., 2010) and chimpanzee ( Duval et al., 2010; Krief et al., 2010), *P. vivax* was from African apes ( Liu et al., 2014), *P. malariae* was from chimpanzees ( Duval et al., 2010) and *P. knowlesi* was from monkeys ( Knowlesi, 1935; Zhang et al., 2016), while *P. ovale* in humans and chimpanzees are genetically identical ( Duval et al., 2009). The factors hypothesized to explain this situation include primate’s habitat loss and human’s aggressiveness in exploring forest ( Davidson et al., 2019). A study from South Kalimantan reported the contribution of forest workers to malaria incidence ( Rahayu et al., 2016). Hence, this research was conducted in Gaura village, West Sumba and Fakfak, West Papua which has a high percentage of forestry workers.

East Nusa Tenggara and West Papua are known as malaria-endemic province areas in Indonesia as their annual parasite incidence (API) in 2015 was 31.29% and 7.04%, respectively ( Kemenkes, 2016), while in 2018 was 8.49% and 3.42%, respectively. Even though there was a decrease in API numbers, according to the health office in both the districts, the API rate in Fakfak, West Papua and East Nusa Tenggara, West Sumba still high cases, which have 4.85% and 12.9%, respectively ( Public Health Office of Fakfak and West Sumba, unpublished data). The trend of malaria cases in each region is different, although reports in several provinces have decreased, both areas still have a high malaria incidence. Due to this situation, we aimed to explore the presence of human *Plasmodium* among domestic animals that are a potential reservoir host in high malaria transmission areas. Therefore we can ascertain one of the reasons why these areas still has a high endemicity rate.

## Methods

### Study area and population

**Figure 1.**
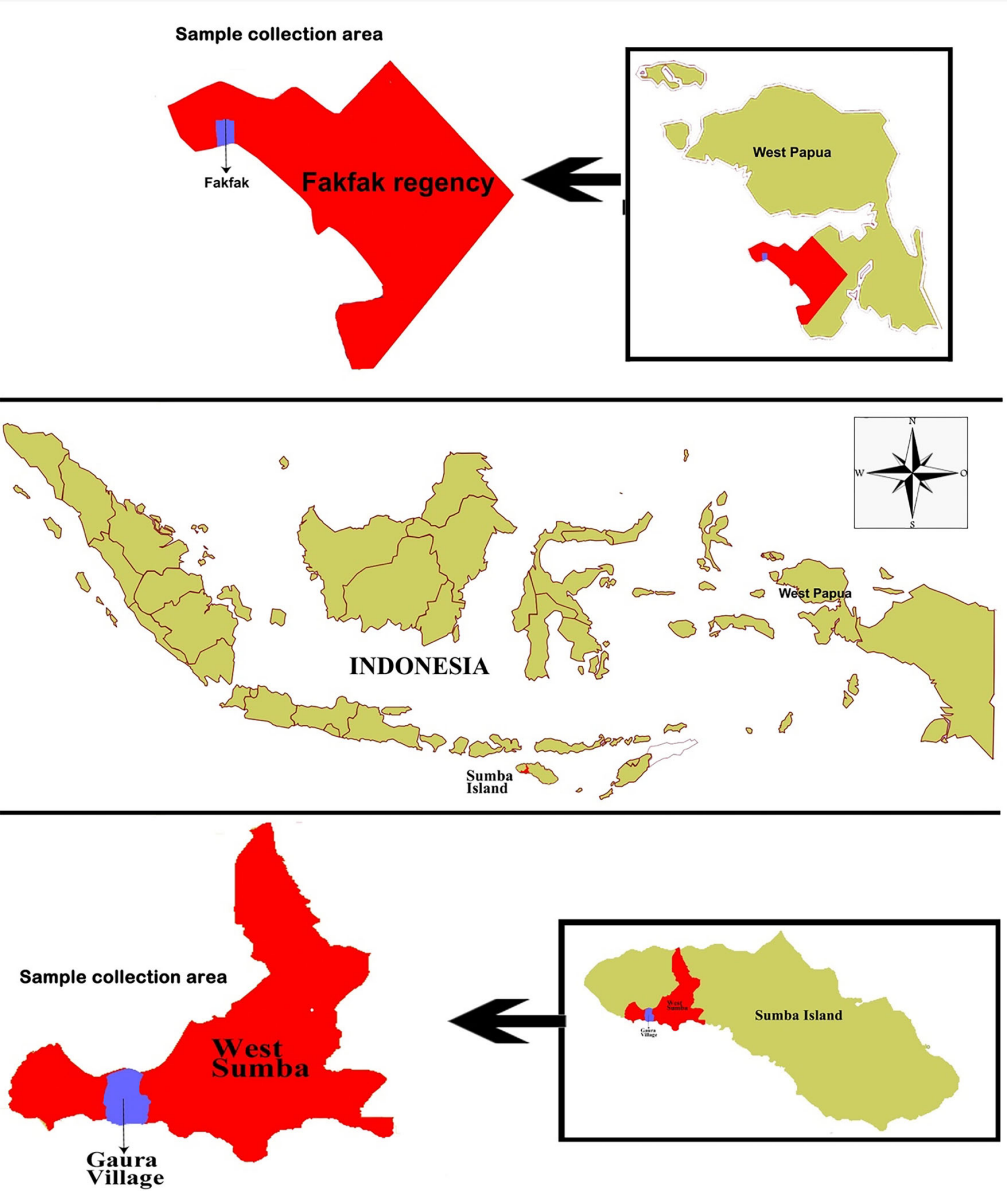
Study area: Map of Gaura Village, West Lamboya, West Sumba, Indonesia and Fakfak Regency, West Papua, Indonesia.

This study was conducted in October 2018 in Gaura village, West Sumba Regency, an area 29.96 km ^2^ in size inhabited by 9,584 people, and Fakfak, West Papua Province, in August 2019 with an area of 11,036 km ^2^ inhabited by 84,692 people ( Figure 1). The residents’ main occupation is farming, while livestock such as goats, horses, cows, pigs, and buffalos are commonly found in their enclosures located around the owner’s residence. Furthermore, they also own pets such as dogs and cats. The average distance between enclosure and home in Fakfak was 225 meters while in Gaura village the distance was 0-10 meters.

### Sample collection

Sampling was carried out by the veterinarian and staff from West Sumba and Fakfak Animal Husbandry Office. The buffaloes, goats, pigs, and horses’ blood samples were collected in 5 ml EDTA tubes from the jugular vein located in the ventrolateral area of the neck using vacutainer needles, size 16–18. Meanwhile, the dog’s blood was drawn from *the cephalic antebrachial* vein in the leg using a size 21 vacutainer needle. By using a sterile micropipette, approximately 10 ul of EDTA blood was dropped onto a microscope slide, then smeared and stained with Giemsa (MERCK Millipore, Germany) for *Plasmodium* microscopic identification, while the remaining blood was dropped by sterile micropipet (about 20 ml) onto a filter paper (Whatman CAT No. 1442-090) until it absorbed to about 1.5 cm in diameter *and hold back until blood at filter paper dry.* The dry filter paper was put on a sterile clip seal plastic bags and stored at room temperature for a maximum of 10 days. All the sample collection process was done by sterile conditions.

### DNA extraction

A dried blood spot (DBS) isolation kit for DNA extraction on filter paper (Cat. no. 36000) from Norgen Biotec was used. A 6 × 3 mm piece of blood-stained filter paper was put into a 1.5 ml tube containing 100 μl of digestion buffer B. It was vortexed and incubated at 85°C for 10 minutes. Afterwards, 20 μl of proteinase K and 300 μl of lysis buffer B were added to the tube and then vortexed before incubation at 56°C for 10 minutes. About 250 μl of 95% ethanol was added to the tube and then vortexed, while the DNA content was washed by adding 500 μl of WN wash solution and centrifugated for one minute at 8,000 rpm. Washing was carried out again using 500 μl of WN wash solution and centrifugated at 14,000 *rpm.* For DNA elution, 90 μl of elution buffer B was put into the tube and centrifuged at 8,000 rpm for one minute, and the purified DNA was stored at -20°C.

### DNA amplification and electrophoresis

DNA amplification by nested PCR and qPCR were performed as directed by Tiangen Biotech (Beijing). *Plasmodium* DNA amplification was carried out using the nested PCR method with a 2× Tag Plus PCR mix enzyme (Tiangen). The final volume of 12.5 μl contained 6.25 μl enzyme, 2.25 μl ddH _2_O, 1 μl forward primers, 1 μl reverse primers, and 2 μl DNA sample. For sequencing, the PCR mixture’s volume was doubled, with the final volume being 25 μl, while the primer sequences of *P. falciparum*, *P. vivax* ( Snounou et al., 1993a) and *P. knowlesi* ( Lee et al., 2011) can be seen in Table 1.

**Table 1.** Primer sequences for nested PCR.

Nested PCR	Species	Sequences primers	Size (bp)
Nested 1	*Plasmodium*	rPLU6: 5′-TTAAAATTGTTGCAGTTAAAACG-3′ rPLU5: 5′-CCTGTTGTTGCCTTAAACTTC-3	1200
Nested 2	*P. falciparum*	rFAL1: 5′-TTAAACTGGTTTGGGAAAACCAAATATATT-3′ rFAL2: 5′-ACACAATGAACTCAATCATGACTACCCGTC-3′	205
Nested 2	*P. vivax*	rVIV1: 5′-CGCTTCTAGCTTAATCCACATAACTGATAC-3′ rVIV2: 5′-ACTTCCAAGCCGAAGCAAAGAAAGTCCTTA-3′	120
Nested 2	*P. knowlesi*	Kn1f: 5′-CTCAACACGGGAAAACTCACTAGTTTA-3′ Kn3r: 5′-GTATTATTAGGTACAAGGTAGCAGTATGC-3′	296
Other primers	*P. falciparum*	rPF1: 5′-AGAAATAGAGTAAAAAACAATTTA-3′ rPF2: 5′-GTAACTATTCTAGGGGAACTA-3′	918
Other primers	*P. vivax*	rPV1: 5′-CCGAATTCAGTCCCACGT-3′ rPV2: 5′-GCTTCGGCTTGGAAGTCC-3′	714

The nested one DNA amplification temperature was set at 94°C denaturation (one minute), 55°C annealing (one minute) and 72°C extension (one minute) for 35 cycles. For nested two, denaturation was carried out at 94°C (30 seconds), 55°C annealing (one minute) and extension was at 72°C (30 seconds) in 35 cycles. There was a difference in the annealing temperature for each species in nested two, namely 55°C (one minute) for PCR multiplex *P. falciparum* and *P. vivax*, but 56°C (one minute) for *P. knowlesi.* Nested one products were used as templates for nested two and both were run on agarose gel 1.5% and 2%, respectively, while qPCR was run on agarose gel 1.5% and view in a gel documentation system. All the stage of DNA amplification was carried out in sterile media and places such us laminar airflow. Molecular work was not performed for *P. ovale* and *P. malariae* due to difficulties in finding the positive control, and according to the local health office these species have never been reported from Sumba and Fakfak.

Preventing the possibility of positive contamination of DNA *Plasmodium* in the first extraction, DNA was re-extracted from the same blood spot at filter paper. The extraction room used was confirmed to have never been used for *Plasmodium* extraction sample previously. Filter papers were cut by scissors which has been sterilization. PCR was performed using the primers, rPF1 and rPF2, as well as rPV1 and rPV2 ( Snounou et al., 1993b) to detect *P. falciparum* and *P. vivax*, respectively. The same extraction and amplification method were used as described above.

### Sequencing and alignment

To determine the *Plasmodium* species, in the second round of nested PCR, products having positive band targets were sent to the 1 ^st^ BASE, Axil Scientific Pte Ltd Singapore for sequencing. The DNA sequence result was adjusted using multiple alignments found in the BioEdit 7.0 application ( Hall et al., 2011) and then read by the BLAST program from the NCBI website.

### Ethical clearance

This study was approved for ethical clearance by the ethics committee of the Faculty of Medicine, Hasanuddin University (734/H4.8.4.5.31/PP36-KOMETIK/2018). All efforts were made to ameliorate any suffering of animals. To prevent stress, animals were comforted by their owners while blood samples were taken, and sampling was performed by experienced officers. Second and third blood samples were taken if there was a failure in the first sample and only if the animals were cooperative. About 20% of animals were sampled more than once.

### Results

**Figure 2.**
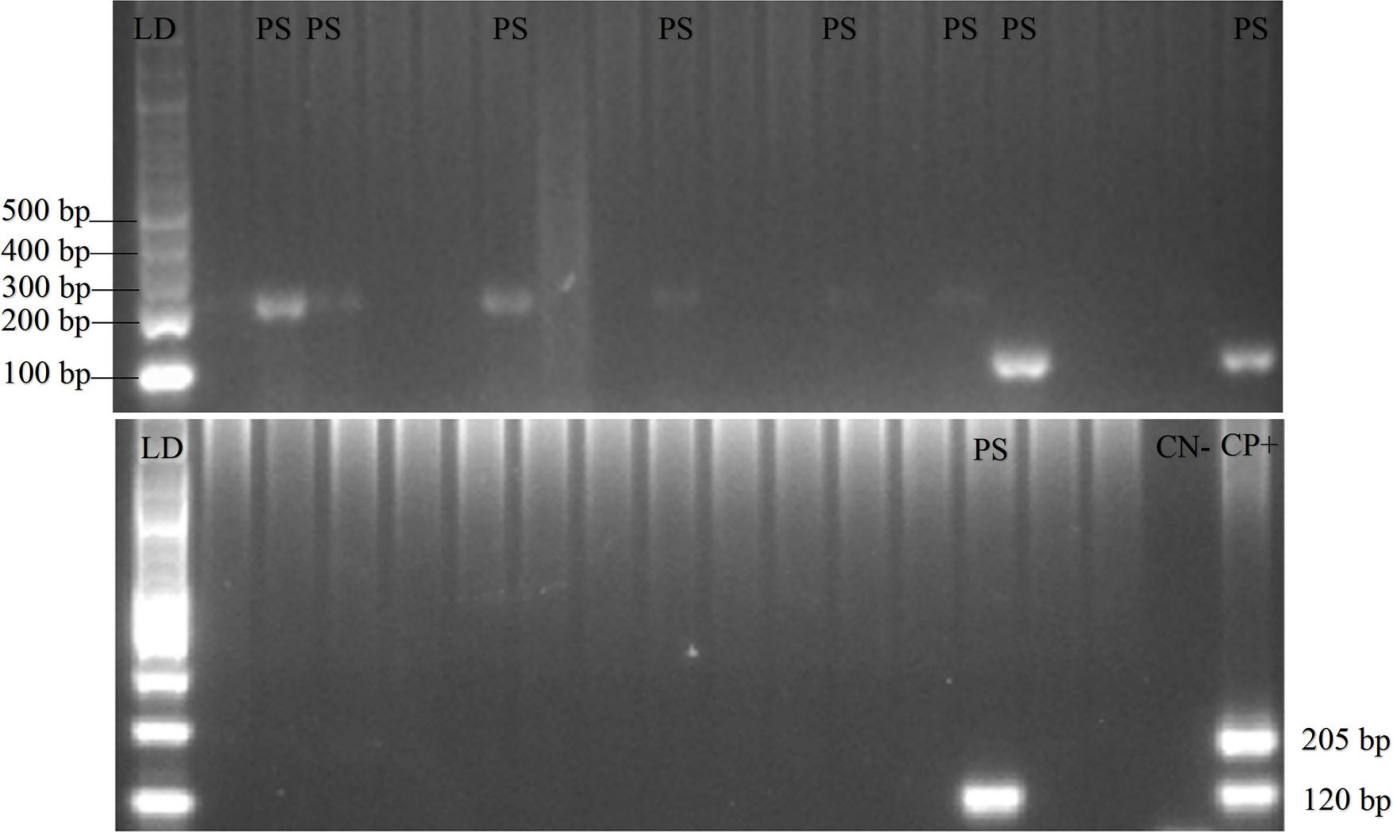
Gel view of PCR product from *Plasmodium vivax* and *Plasmodium falciparum* in domestic animals in Gaura, West Sumba (LD = DNA ladder, PS = positive samples, CN = control negative, CP = control positive) by nested PCR (multiplex PCR). 120 bp for positive *Plasmodium vivax*, 205 bp for positive *Plasmodium falciparum.*

**Figure 3.**
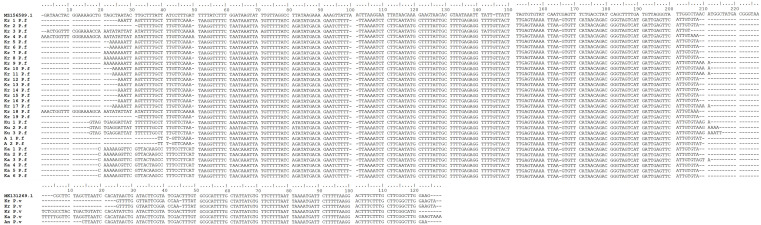
*Plasmodium falciparum* and *P. vivax* DNA sequence alignments from blood samples taken in Gaura village, West Sumba, Indonesia by ClustalW multiple sequence alignment (Kr = buffalo, Ku = Horse, A/An = dog, Ka = goat, P.f = *P. falciparum*, P.v = *P. vivax*).

**Figure 4.**
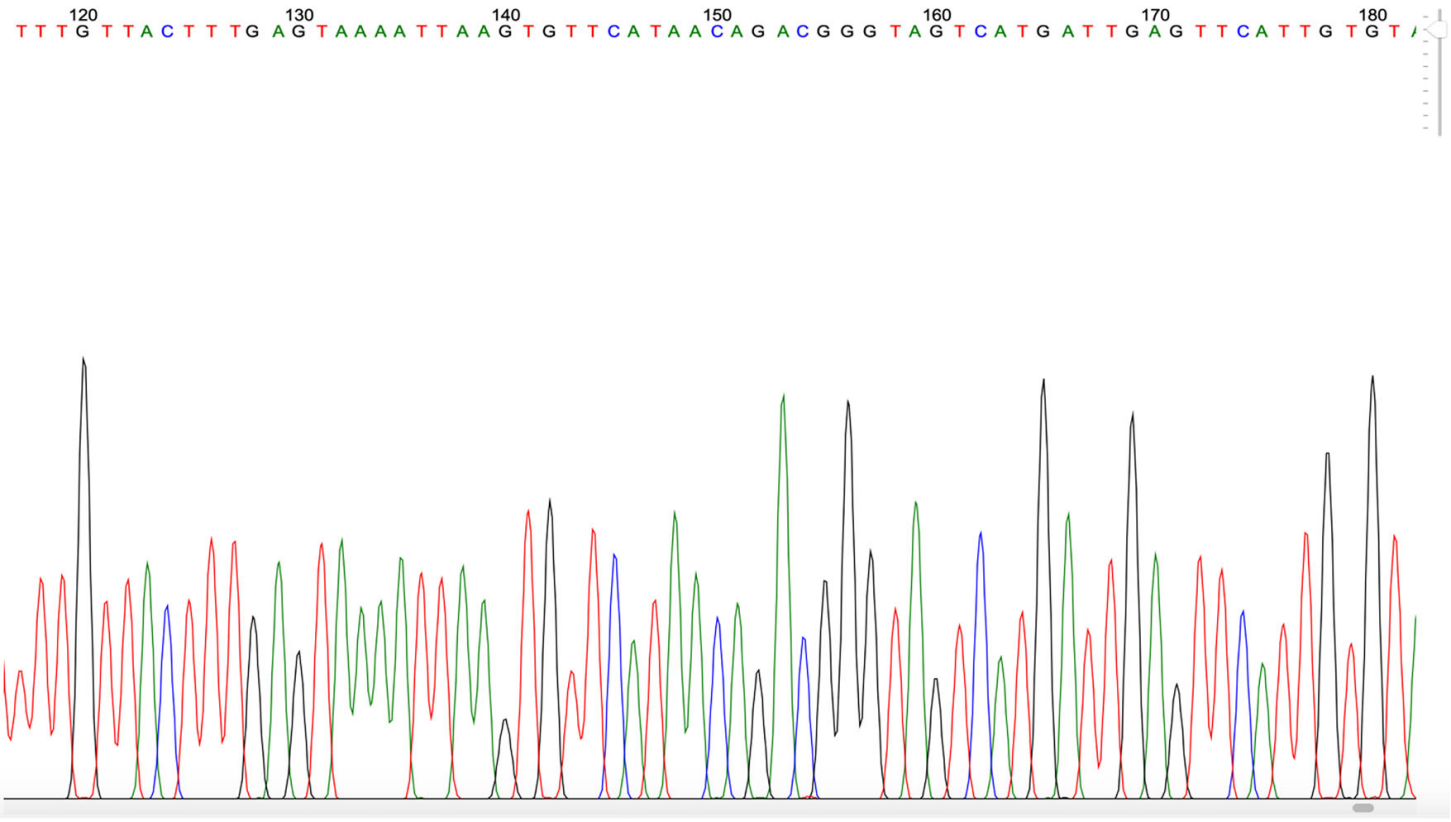
Example of *Plasmodium* PCR product quality from a blood sample taken in Gaura village, West Sumba, Indonesia.

A total of 208 and 62 animal blood samples were collected from Gaura and Fakfak villages, respectively. These consisted of 92 buffalos, 21 horses, 121 goats, 18 dogs, and 18 pigs. Using the nested PCR method, 32 of the 270 animals were found to be *P. falciparum* and *P. vivax* positive *.* The percentage of *Plasmodium* positive animals included 20.7% buffalo, 14.3% horse, 5.8% goat, and 16.7% dog with one buffalo having a mixed infection ( *P. falciparum* and *P. vivax*). There was no *P. knowlesi* found in any of the samples and no any form of malaria parasites was found in 18 pig blood samples. PCR gel products, DNA sequence results, and the sample’s quality can be seen in Figures 2, 3 and 4, respectively ( Munirah et al., 2021). *Plasmodium* distribution in the animals’ blood samples from Gaura and Fakfak are presented in Table 2, and it shows that blood containing *Plasmodium* was only found in Gaura. The results of the qPCR using rPF1–rPF2 and rPV1–rPV2 primers were similar to the nested PCR.

**Table 2.** Distribution of animal blood samples and *Plasmodium* species found in Gaura village, West Sumba, Indonesia and Fakfak, West Papua, Indonesia.

No	Domestic animals	Number of samples		Positive *Plasmodium*				Total positive
		Gaura (2018)	Fakfak (2019)	Pf	Pv	Pk	Mix Pf & Pv	
1	Buffalos ( *Bubalus bubalis)*	92	0	15	3	0	1	19
2	Horse ( *Equus caballus*)	21	0	3	0	0	0	3
3	Goat ( *Capra aegagrus hircus*)	72	49	6	1	0	0	7
4	Dog ( *Canis lupus familiaris*)	10	8	2	1	0	0	3
5	Pig ( *Sus scrofa domesticus*)	13	5	0	0	0	0	0
	**Total**	**208**	**62**	**26**	**5**	**-**	**1**	**32**

Note: Pf = *P. falciparum*, Pv = *P. vivax*, Pk = *P. knowlesi*.

**Figure 5.**
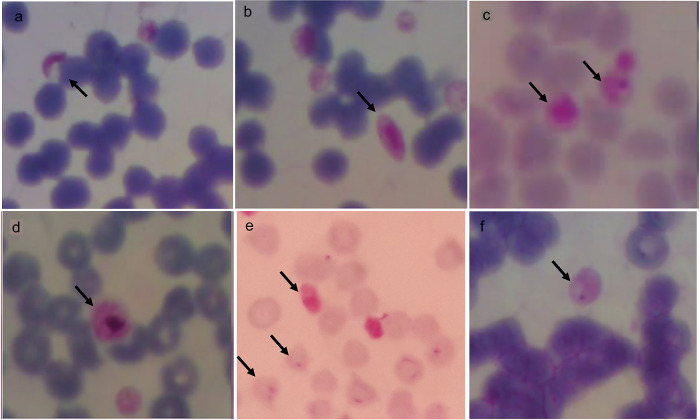
Morphology of *Plasmodium* in animals from Gaura village, West Sumba, Indonesia. Gametocytes (a,b,d) in buffalo, schizont in horse (c), gametocyte and trophozoite in goat (e) and trophozoite in horse (f) with magnification 1000 ×.

Microscopically, trophozoites, schizonts, and gametocyte forms at 100× magnification can be seen in Figure 5. *P. falciparum* gametocytes found in buffaloes were sausage and crescent-shaped (a, b), while schizonts found in horses were smaller or the same size as the red blood cells (c). The *P. vivax* gametocyte was larger than the red blood cells found in buffalo (d). *P. falciparum* gametocyte and trophozoites (ring-shaped) with one or two nuclei was found in goats (e) and *P. falciparum* trophozoite found in horses had one nucleus (f).

### Discussion

The presence of *Plasmodium* was suspected in domestic animals because malaria cases in these two villages remained high despite maximum preventive efforts having been applied including insecticide-treated bed nets. About 32 of the 270 blood (11.9%) samples contained human *Plasmodium* parasites, and this is the first data report and further study is therefore needed.

Previous studies found *Plasmodium relictum* in avian species ( Cox, 2010), *P. cephalophi* in ungulates ( Bruce et al., 1913), *P. traguli* in mousedeer ( Garnham and Edeson, 1962), *P. brucei* in gray duiker ( Bruce et al., 1915; Templeton et al., 2016a), *P. bubalis* in water buffalo ( Sheather, 1919), and *P. odocoilei* in white-tailed deer ( Garnham and Kuttler, 1980; Perkins and Schaer, 2016). Other parasites found included *P. caprae* in goats (ruminant) ( Kaewthamasorn et al., 2018), *P. bergei in* Rodentia ( Vincke and Lips, 1948), and *P. cynomolgi*, *P. inui*, and *P. fragile* in primates ( Dixit et al., 2018). The five *Plasmodium* species that infect humans were originally parasites in primates ( Duval et al., 2010; Knowlesi, 1935; Liu et al., 2014; Ng et al., 2008; Singh and Daneshvar, 2013; Zhang et al., 2016). In this study, *P. falciparum* was found in buffalos, goats, dogs, and horses, while *P. vivax* was in buffalos, goats, and dogs. Initially, the presence of *Plasmodium* in these animals’ erythrocytes was not certain. However, the nested PCR showed the same results for all positive samples. The sequencing results of the positive bands in the nested PCR two analysis showed the bands were *P. falciparum* and *P. vivax* ( Figure 3). The number of positive *Plasmodium* in buffaloes is higher because these animals are susceptible to parasitic infections. As reported by Mursyid who found as many as 10 parasitic infections in buffalo in Central Lombok ( Mursyid et al., 2020). In addition, other studies declare that buffalo ages is one of the factors cause a high risk of parasite infection ( Nurhidayah et al., 2019). It is known that the average age of buffaloes is more than 7 years. This is the first investigation reporting human *Plasmodium* in domestic animals (ruminant, ungulate, and carnivore).

Studies conducted by Templeton, in Thailand, in 2008 and 2015 found *P. bubalis* in buffalo. Microscopic appearance of *P. bubalis* can be seen in the journal Templeton et al. (2016b). Through this figure, there is no similarity between *P. bubalis* and *P. falciparum* gametocytes that found in buffaloes in our study. Furthermore, in Thailand, Myanmar, Iran, Kenya and Sudan, using molecular examination methods targeting the cytochrome b (cytb) gene found *P. caprae* in goats. The trophozoites of *P. caprae* can be seen in a journal published by Kaewthamasorn et al. (2018).

*Plasmodium* discovery among domestic animals in malaria-endemic areas raises the following questions. How do *P. falciparum* and *P. vivax* live in these animals? Are they intermediate hosts for this parasite? Did these *Plasmodium* species evolve to live in ruminants, ungulates, and carnivores? As a result of repeated exposure, have these animals become more permissive to *Plasmodium*, which generally lives in humans? Is this parasite pathogenic in animals? *P. knowlesi* is a commensal microbe in primates but pathogenic in humans ( Jongwutiwes et al., 2004; Ng et al., 2008; Singh and Daneshvar, 2013) and its migration from primates to humans is caused by forest loss or human invasion of primate habitat ( Davidson et al., 2019). There is a possibility that animal and human proximity aids easier cross parasite transfer between both groups by mosquitoes. In humans, both parasites infect by growing first in liver cells and moving to red blood cells. The parasites multiplicate in red blood and lead to medical conditions characterized by fever, chills, headache, profuse sweating, weakness, rheumatic pain, symptoms of anemia or lack of blood and nausea or vomiting. Until now it is undetermined whether the *Plasmodium* parasites found in non-primate animals are pathogenic or commensal. Nevertheless, *P. brasilianum* found in animals, already identified that the sporozoites migrate directly to the liver and multiply itself then release merozoites. The merozoites break out infect erythrocytes then cause symptomatic disease in these animals ( Erkenswick et al., 2017). Infected organism by *Plasmodium* ordinarily malignant if the cycle of parasites advanced to erythrocyte stage or causes malaria due to the rupture of red blood cells. The data regarding intermediate hosts of *P. falciparum* and *P. vivax* in livestock are still a challenge to verified. Furthermore, various studies have attempted to provide evidence that these two *Plasmodium* originated from chimpanzees and gorillas.

The research has conducted by Prugnolle in 2013 stated that there is a link between *P. vivax* in monkeys and humans. The results declare a possibility of natural transfer from humans to apes and vice versa, particularly if animals were around humans which permit transfer of parasites continuously through vectors ( Prugnolle et al., 2013). Another study by Mu in 2005 concluded that *P. vivax* is a zoonotic parasite ( Mu et al., 2005).

Despite high API in Fakfak and Gaura village, West Sumba, only the animals from Gaura village in West Sumba had human *Plasmodium.* This difference is possibly due to the distance between the residents’ houses and animal enclosures as the enclosures are located approximately 50–500 meters from the main houses in Fakfak. Meanwhile, in Gaura, residents live in stilt houses where the ground floor functions as an animal shelter and around the house, allowing microbial transfer between humans and animals by mosquitoes. In Fakfak, the sampling locations were not easily accessible, and the steep geographical conditions made it difficult to collect many samples compared to Gaura. We conducted a census of vector to both areas. In Gaura village, West Sumba we collected 11 Anopheles namely *An. vagus*, *An. sundaicus*, *An. aconitus*, *An. Kochi*, *An. flavirostris*, *An. indefinitus*, *An. maculatus*, *An. minimum*, *An. annularis*, *An. nivipes*, and *An. subpictus.* The molecular examination result show 2 *An. sundaicus* were infected by *Plasmodium*, whereas in Fakfak did not find any *Anopheles.* In India *An. sundaicus* was reported as zoophilic ( Vidhya et al., 2019), whereas in Mekong, Vietnam was reported as more anthropophilic ( Trung et al., 2005). The different reports each region regarding behavior of *An. sundaicus* indicated the reference diversity of the way mosquitoes bited rely on several factors, one of the factors is environmental circumstances. The potential bited *An. sundaicus* in both animals and humans in Gaura village are possible cause the distance proximity of domestic animals and humans. Nevertheless, it needs further research.

Application of livestock for zooprophylaxis in malaria endemic areas has been widely used because it has several advantages. But undeniable it can also increase the survival of mosquitoes. As it is known, longer life mosquitoes have a high potential to become a vector of disease. One of the most significant factors driving parasite transmission is the close proximity between infected and uninfected organism ( Kuris et al., 1980). Research by Hasyim concluded that livestock in malaria endemic areas has a high potential to increase the incidence of malaria in Indonesia ( Hasyim et al., 2018). Zoopotentiation frequently take place when livestock are kept indoors or around houses ( Donnelly et al., 2015). Location setting of livestock a long way from humans will reduce malaria cases ( Franco et al., 2014). Anticipated strategy malaria transmission is separate livestock from humans in sufficient distance to minimize the same mosquito bite between humans and animals. Mosquitoes have obtained animal blood will not seek blood humans anymore. If animal presence is close enough, the pathogen transmission between humans and animals will be massive through intermediary vectors. Zoonoses potential drive by several factors that must be solved including vectors abundance, mosquitoes survival, mosquitoes behavior and other factors such local environment.

Although *Plasmodium* can be detected microscopically due to erythrocyte size, which is smaller in animals than humans, molecular methods become significant in detecting *Plasmodium* presence. The nested PCR was used to detect *Plasmodium* because its sensitivity was equally as high as Real-Time PCR and the cost was relatively lower ( Green and Sambrook, 2019; Perandin et al., 2004). The microscopic method of double fluorescent dye utilization with Giemsa stain is recommended for further studies ( Guy et al., 2007).

Other research is required to confirm domestic animal *Plasmodium* by amplifying the cytochrome B sequence and sequencing the invasion ligands such as DBL protein as well as other investigations that emerge from the results of this study. This is necessary to address various challenges in malaria elimination.

## Conclusion

This study found *Plasmodium* species in domestic animals that previously known as human parasites. These results may partly answer the question of why malaria is challenging to eliminate in an area. It is hoped that these findings can be applied to improve public health and become a reference in formulating malaria prevention strategies.

## Data availability

### Underlying data

Figshare: Underlying data for ‘The discovery of human *Plasmodium* among domestic animals in West Sumba and Fakfak, Indonesia’, https://doi.org/10.6084/m9.figshare.14703012.v3 ( Munirah et al., 2021).

This project contains the following underlying data:

• Gel photo: Result of rPF1–RPF2 primers
• Gel photo: Result of RPV1–rPV2 primers
• Gel photo: Nested PCR *P. falciparum* and *P. vivax*

### Reporting guidelines

*Figshare*: ARRIVE checklist for ‘The discovery of human *Plasmodium* among domestic animals in West Sumba and Fakfak, Indonesia’, https://doi.org/10.6084/m9.figshare.14703012.v3 ( Munirah et al., 2021).

Data are available under the terms of the Creative Commons Attribution 4.0 International license (CC-BY 4.0).
