# The discovery of human *Plasmodium* among domestic animals in West Sumba and Fakfak, Indonesia

**DOI:** 10.12688/f1000research.53946.3

**Published:** 2024-04-09

**Authors:** Munirah Munirah, Sitti Wahyuni, Isra Wahid, Firdaus Hamid

**Affiliations:** 1 Doctoral Study Program, Faculty of Medicine, Hasanuddin University, Makassar, South Sulawesi, 90245, Indonesia; 2 Department of Parasitology, Faculty of Medicine, Hasanuddin University, Makassar, South Sulawesi, 90245, Indonesia; 3 Department of Microbiology, Faculty of Medicine, Hasanuddin University, Makassar. Jln. Perintis Kemerdekaan 10 Tamalanrea, Makassar, South Sulawesi, 90245, Indonesia

**Keywords:** Plasmodium falciparum, Plasmodium vivax, malaria, animals, host reservoir, PCR.

## Abstract

**Background:**

In Indonesia, malaria incidence is at a high rate despite maximum preventive efforts. Therefore, this study aims to determine the possibility of a *Plasmodium* reservoir among domestic animals in malaria-endemic areas.

**Methods:**

Animal blood was collected using EDTA tubes, then smeared and stained with Giemsa for *Plasmodium* microscopic identification. About 10 μl of blood was dropped on to a filter paper to capture *Plasmodium* DNA. Nested PCR was used for parasite molecular detection, while *Plasmodium* species were identified using the sequenced DNA.

**Results:**

A total of 208 and 62 animal blood samples were collected from Gaura village, West Sumba and Fakfak village, West Papua, Indonesia respectively. In total, 32 samples from Gaura contained *P. falciparum* or *P. vivax*, while the *Plasmodium* percentage in buffalo, horse, goat, and dogs were 20.7%, 14.3%, 5.8%, 16.7%, respectively. *P. knowlesi* was not found in any of the samples, and no other species were detected in 18 pig blood samples.

**Conclusion:**

Human *Plasmodium* existence among domestic animals in Indonesia partly explains the high prevalence and persistence of malaria in some endemic areas due to a reservoir host presence. Therefore, future studies need to ascertain the cause.

## Introduction

Indonesia is striving toward becoming a malaria-free country through the Malaria Control Programme. Despite the implementation of several efforts, such as early detection, treatment, and mosquito vector eradication, the disease remains a persistent challenge. Factors including parasite resistance to antimalarial drugs, mosquito resistance to insecticides, inadequate health system performance, and host reservoir presence contribute to continuous malaria prevalence.

*Plasmodium falciparum* and *P. vivax* commonly infect humans, leading to high morbidity and mortality. Only humans were considered the primary host for *Plasmodium* species including *P. falciparum, P. vivax, P. malariae,* and *P. ovale* before molecular diagnostics development *.* However, studies over the past two decades reported that the parasites originated from animals *.* Specifically, *P. falciparum* was traced back to gorillas ( Liu et al., 2010) and chimpanzees ( Duval et al., 2010; Krief et al., 2010), while *P. vivax* was associated with African apes ( Liu et al., 2014). Other sources stated were the origination of *P. malariae* from chimpanzees ( Duval et al., 2010) and *P. knowlesi* from monkeys ( Knowlesi, 1935; Zhang et al., 2016), while *P. ovale* found in humans and chimpanzees had genetic similarities ( Duval et al., 2009). Factors such as habitat loss among primates and human encroachment into forested areas are believed to have facilitated the transmission of these parasites ( Davidson et al., 2019). An investigation performed in South Kalimantan reported the contribution of forest workers to malaria incidence ( Rahayu et al., 2016). Therefore, this study was conducted in Gaura village, West Sumba and Fakfak, West Papua, which contain a high percentage of forestry workers.

West Papua and East Nusa Tenggara are known as malaria-endemic provinces in Indonesia, with annual parasite incidence (API) of 7.04% and 31.29%, respectively in 2015 ( Kemenkes, 2016), while the rates were 3.42% and 8.49% in 2018. Even though there was a decrease in API numbers, Fakfak, West Papua and East Nusa Tenggara, West Sumba, continued to report high API rates of 4.85% and 12.9%, respectively ( Public Health Office of Fakfak and West Sumba, unpublished data). The trend of malaria cases varies, and despite reduced API rates recorded in several provinces, the two areas still experience a high incidence. Therefore, this study aimed to explore the presence of human *Plasmodium* among domestic animals, which might serve as a potential reservoir host in endemic areas. The exploration would provide insights into the reasons for high endemicity rates in the examined areas.

## Methods

### Study area and population

**Figure 1.**
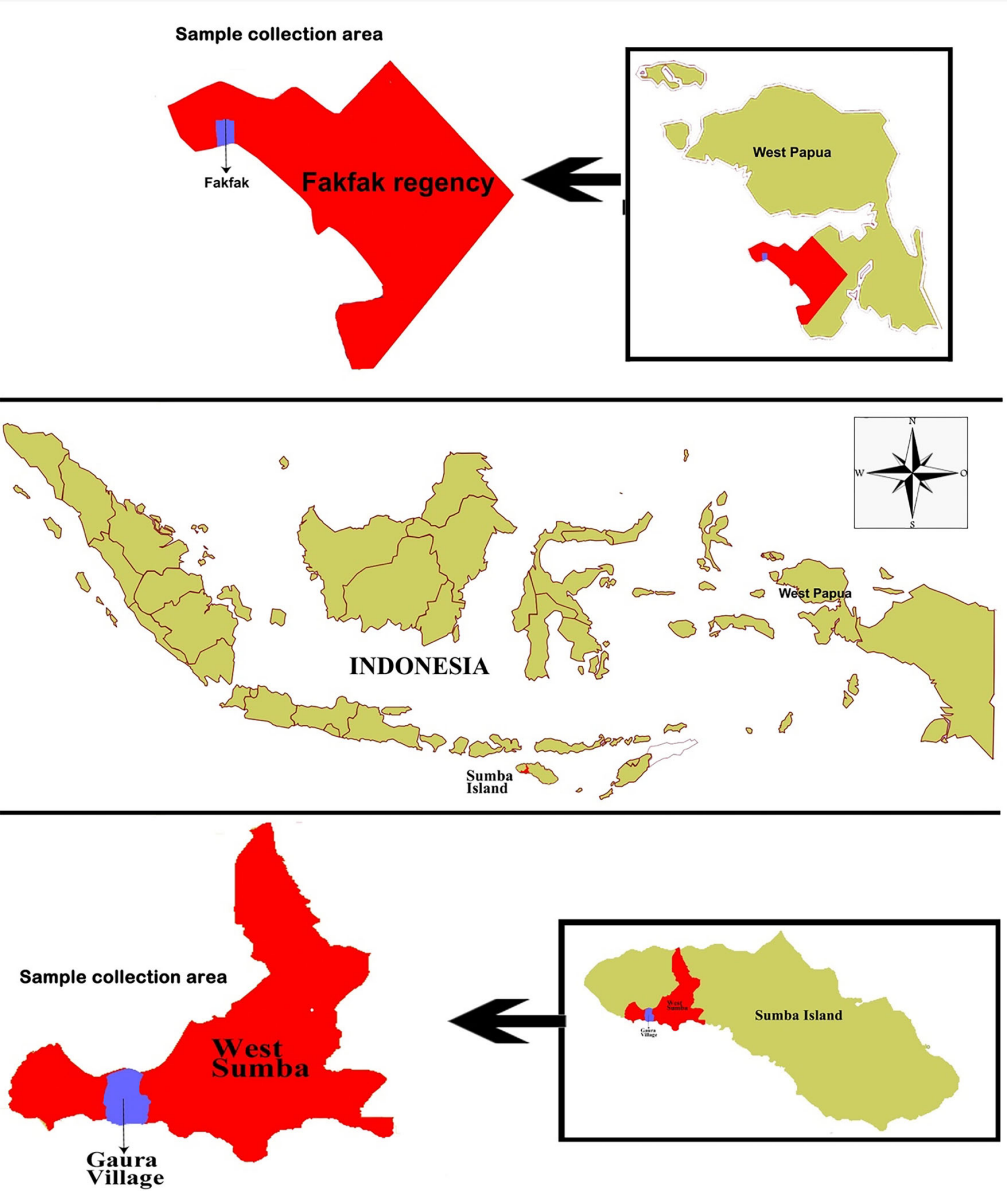
Study area: Map of Gaura Village, West Lamboya, West Sumba, Indonesia and Fakfak Regency, West Papua, Indonesia.

This study was conducted in October 2018 in Gaura village, West Sumba Regency, an area 29.96 km ^2^ in size inhabited by 9,584 people, and Fakfak, West Papua Province, in August 2019 with an area of 11,036 km ^2^ inhabited by 84,692 people ( Figure 1). The residents’ main occupation is farming, while livestock such as goats, horses, cows, pigs, and buffalos are commonly found in their enclosures located around the owner’s residence. Furthermore, they also own pets such as dogs and cats. The average distance between enclosure and home in Fakfak was 225 meters while in Gaura village the distance was 0-10 meters.

### Sample collection

Sampling was carried out by the veterinarian and staff from West Sumba and Fakfak Animal Husbandry Office. The buffaloes, goats, pigs, and horses’ blood samples were collected in 5 ml EDTA tubes from the jugular vein located in the ventrolateral area of the neck using vacutainer needles, size 16–18. Meanwhile, the dog’s blood was drawn from *the cephalic antebrachial* vein in the leg using a size 21 vacutainer needle. By using a sterile micropipette, approximately 10 ul of EDTA blood was dropped onto a microscope slide, then smeared and stained with Giemsa (MERCK Millipore, Germany) for *Plasmodium* microscopic identification, while the remaining blood was dropped by sterile micropipet (about 20 ml) onto a filter paper (Whatman CAT No. 1442-090) until it absorbed to about 1.5 cm in diameter *and hold back until blood at filter paper dry.* The dry filter paper was put on a sterile clip seal plastic bags and stored at room temperature for a maximum of 10 days. All the sample collection process was done by sterile conditions.

### DNA extraction

A dried blood spot (DBS) isolation kit for DNA extraction on filter paper (Cat. no. 36000) from Norgen Biotec was used. A 6 × 3 mm piece of blood-stained filter paper was put into a 1.5 ml tube containing 100 μl of digestion buffer B. It was vortexed and incubated at 85°C for 10 minutes. Afterwards, 20 μl of proteinase K and 300 μl of lysis buffer B were added to the tube and then vortexed before incubation at 56°C for 10 minutes. About 250 μl of 95% ethanol was added to the tube and then vortexed, while the DNA content was washed by adding 500 μl of WN wash solution and centrifugated for one minute at 8,000 rpm. Washing was carried out again using 500 μl of WN wash solution and centrifugated at 14,000 *rpm.* For DNA elution, 90 μl of elution buffer B was put into the tube and centrifuged at 8,000 rpm for one minute, and the purified DNA was stored at -20°C.

### DNA amplification and electrophoresis

DNA amplification by nested PCR and qPCR were performed as directed by Tiangen Biotech (Beijing). *Plasmodium* DNA amplification was carried out using the nested PCR method with a 2× Tag Plus PCR mix enzyme (Tiangen). The final volume of 12.5 μl contained 6.25 μl enzyme, 2.25 μl ddH _2_O, 1 μl forward primers, 1 μl reverse primers, and 2 μl DNA sample. For sequencing, the PCR mixture’s volume was doubled, with the final volume being 25 μl, while the primer sequences of *P. falciparum*, *P. vivax* ( Snounou et al., 1993a) and *P. knowlesi* ( Lee et al., 2011) can be seen in Table 1.

**Table 1.** Primer sequences for nested PCR.

Nested PCR	Species	Sequences primers	Size (bp)
Nested 1	*Plasmodium*	rPLU6: 5′-TTAAAATTGTTGCAGTTAAAACG-3′ rPLU5: 5′-CCTGTTGTTGCCTTAAACTTC-3′	1200
Nested 2	*P. falciparum*	rFAL1: 5′-TTAAACTGGTTTGGGAAAACCAAATATATT-3′ rFAL2: 5′-ACACAATGAACTCAATCATGACTACCCGTC-3′	205
Nested 2	*P. vivax*	rVIV1: 5′-CGCTTCTAGCTTAATCCACATAACTGATAC-3′ rVIV2: 5′-ACTTCCAAGCCGAAGCAAAGAAAGTCCTTA-3′	120
Nested 2	*P. knowlesi*	Kn1f: 5′-CTCAACACGGGAAAACTCACTAGTTTA-3′ Kn3r: 5′-GTATTATTAGGTACAAGGTAGCAGTATGC-3′	296
Other primers	*P. falciparum*	rPF1: 5′-AGAAATAGAGTAAAAAACAATTTA-3′ rPF2: 5′-GTAACTATTCTAGGGGAACTA-3′	918
Other primers	*P. vivax*	rPV1: 5′-CCGAATTCAGTCCCACGT-3′ rPV2: 5′-GCTTCGGCTTGGAAGTCC-3′	714

The nested one DNA amplification temperature was set at 94°C denaturation (one minute), 55°C annealing (one minute) and 72°C extension (one minute) for 35 cycles. For nested two, denaturation was carried out at 94°C (30 seconds), 55°C annealing (one minute) and extension was at 72°C (30 seconds) in 35 cycles. There was a difference in the annealing temperature for each species in nested two, namely 55°C (one minute) for PCR multiplex *P. falciparum* and *P. vivax*, but 56°C (one minute) for *P. knowlesi.* Nested one products were used as templates for nested two and both were run on agarose gel 1.5% and 2%, respectively, while qPCR was run on agarose gel 1.5% and view in a gel documentation system. All the stage of DNA amplification was carried out in sterile media and places such us laminar airflow. Molecular work was not performed for *P. ovale* and *P. malariae* due to difficulties in finding the positive control, and according to the local health office these species have never been reported from Sumba and Fakfak.

Preventing the possibility of positive contamination of DNA *Plasmodium* in the first extraction, DNA was re-extracted from the same blood spot at filter paper. The extraction room used was confirmed to have never been used for *Plasmodium* extraction sample previously. Filter papers were cut by scissors which has been sterilization. PCR was performed using the primers, rPF1 and rPF2, as well as rPV1 and rPV2 ( Snounou et al., 1993b) to detect *P. falciparum* and *P. vivax*, respectively. The same extraction and amplification method were used as described above.

### Sequencing and alignment

To determine the *Plasmodium* species, in the second round of nested PCR, products having positive band targets were sent to the 1 ^st^ BASE, Axil Scientific Pte Ltd Singapore for sequencing. The DNA sequence result was adjusted using multiple alignments found in the BioEdit 7.0 application ( Hall et al., 2011) and then read by the BLAST program from the NCBI website.

### Ethical clearance

This study was approved for ethical clearance by the ethics committee of the Faculty of Medicine, Hasanuddin University (734/H4.8.4.5.31/PP36-KOMETIK/2018). All efforts were made to ameliorate any suffering of animals. To prevent stress, animals were comforted by their owners while blood samples were taken, and sampling was performed by experienced officers. Second and third blood samples were taken if there was a failure in the first sample and only if the animals were cooperative. About 20% of animals were sampled more than once.

## Results

**Figure 2.**
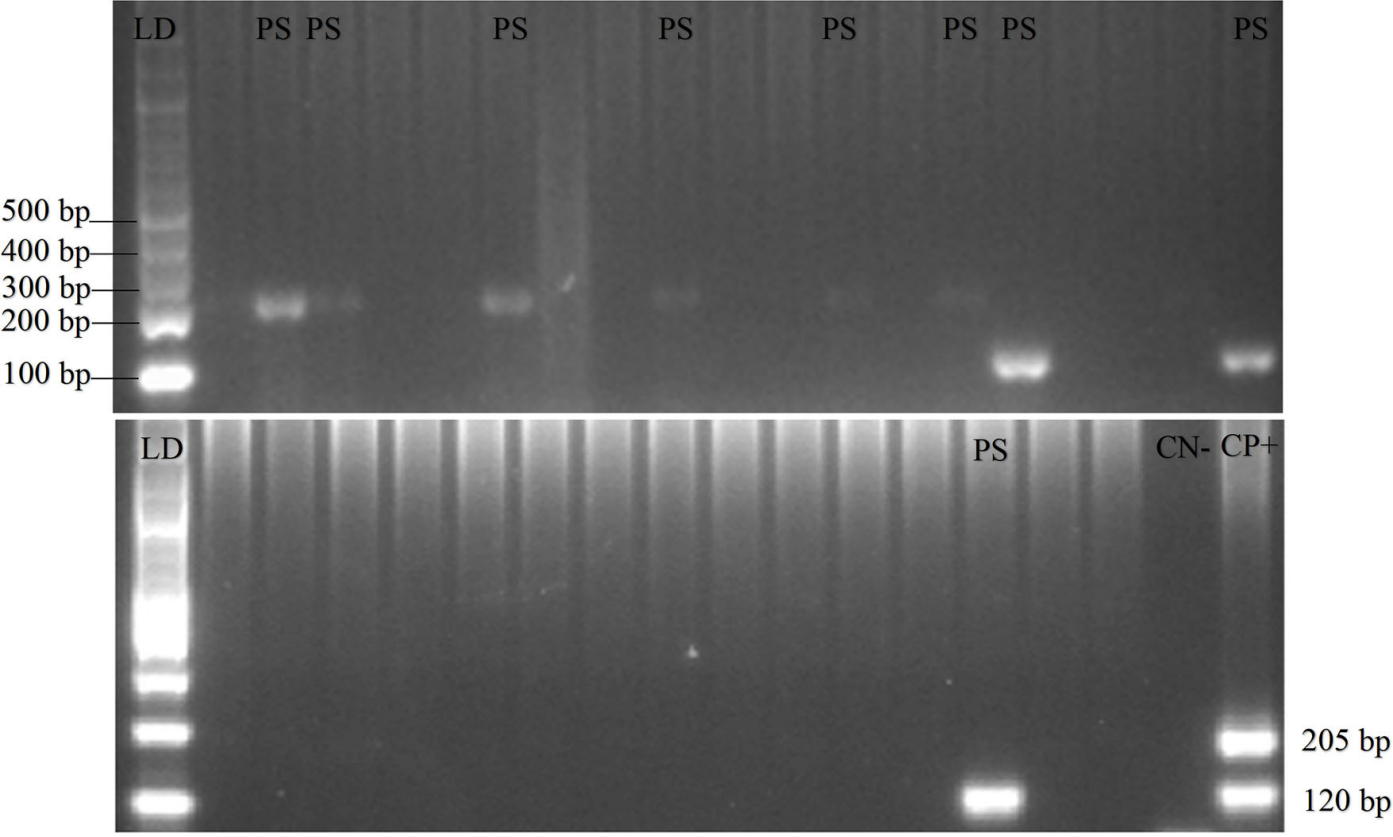
Gel view of PCR product from *Plasmodium vivax* and *Plasmodium falciparum* in domestic animals in Gaura, West Sumba (LD = DNA ladder, PS = positive samples, CN = control negative, CP = control positive) by nested PCR (multiplex PCR). 120 bp for positive *Plasmodium vivax*, 205 bp for positive *Plasmodium falciparum.*

**Figure 3.**
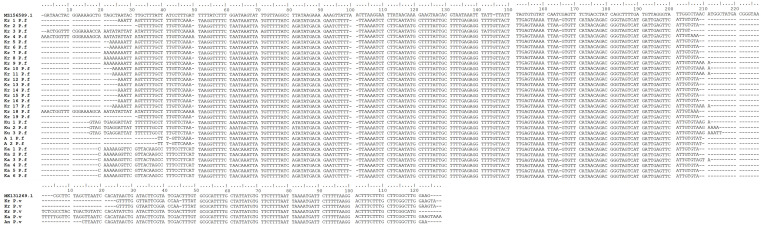
*Plasmodium falciparum* and *P. vivax* DNA sequence alignments from blood samples taken in Gaura village, West Sumba, Indonesia by ClustalW multiple sequence alignment (Kr = buffalo, Ku = Horse, A/An = dog, Ka = goat, P.f = *P. falciparum*, P.v = *P. vivax*).

**Figure 4.**
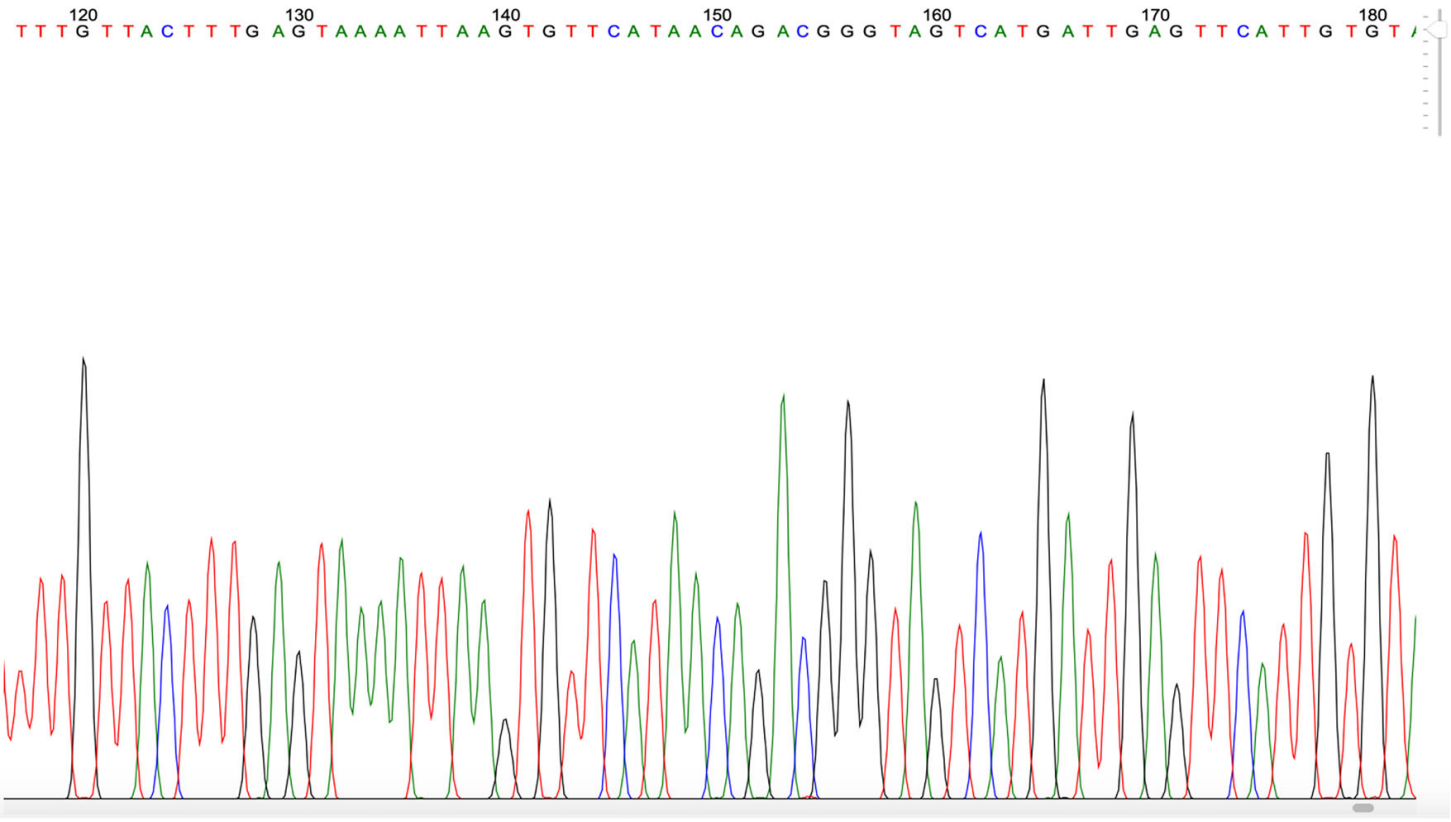
Example of *Plasmodium* PCR product quality from a blood sample taken in Gaura village, West Sumba, Indonesia.

A total of 208 and 62 blood samples were collected in Gaura and Fakfak villages, respectively, from 92 buffalos, 21 horses, 121 goats, 18 dogs, and 18 pigs. Tests conducted using the nested PCR method identified 32 of the 270 animals as positive for *P. falciparum* and *P. vivax.* Furthermore, 20.7% buffalo, 14.3% horse, 5.8% goat, and 16.7% dog were *Plasmodium*-positive, with one buffalo showing mixed infections of *P. falciparum* and *P. vivax.* *P. knowlesi* was absent in all the blood samples and no form of malaria parasites was found in the 18 pigs. Additionally, PCR gel products, DNA sequence results, and the quality of samples can be seen in Figures 2, 3, and 4, respectively ( Munirah et al., 2021). *Plasmodium* distribution in samples from Gaura and Fakfak are presented in Table 2, showing that blood containing the malaria parasites was only found in Gaura. The results of qPCR performed using rPF1–rPF2 and rPV1–rPV2 primers were similar to those of the nested PCR.

**Table 2.** Distribution of animal blood samples and *Plasmodium* species found in Gaura village, West Sumba, Indonesia and Fakfak, West Papua, Indonesia.

No	Domestic animals	Number of samples		Positive *Plasmodium*				Total positive
		Gaura (2018)	Fakfak (2019)	Pf	Pv	Pk	Mix Pf & Pv	
1	Buffalos ( *Bubalus bubalis)*	92	0	15	3	0	1	19
2	Horse ( *Equus caballus*)	21	0	3	0	0	0	3
3	Goat ( *Capra aegagrus hircus*)	72	49	6	1	0	0	7
4	Dog ( *Canis lupus familiaris*)	10	8	2	1	0	0	3
5	Pig ( *Sus scrofa domesticus*)	13	5	0	0	0	0	0
	**Total**	**208**	**62**	**26**	**5**	**-**	**1**	**32**

Note: Pf = *P. falciparum*, Pv = *P. vivax*, Pk = *P. knowlesi*.

**Figure 5.**
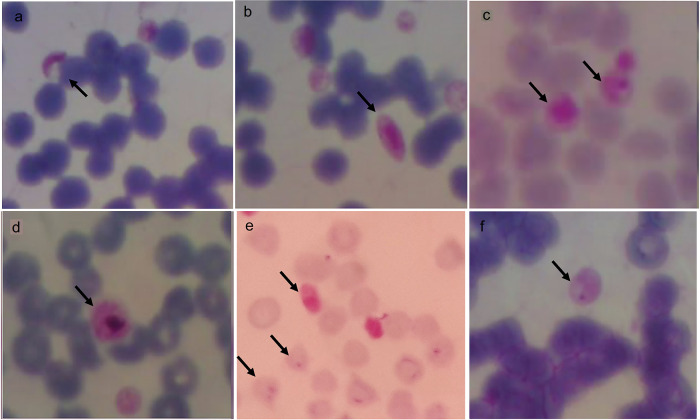
Morphology of *Plasmodium* in animals from Gaura village, West Sumba, Indonesia. Gametocytes (a,b,d) in buffalo, schizont in horse (c), gametocyte and trophozoite in goat (e) and trophozoite in horse (f) with magnification 1000 ×.

Microscopically, trophozoites, schizonts, and gametocyte forms at 100× magnification can be seen in Figure 5. *P. falciparum* gametocytes found in buffaloes were sausage and crescent-shaped (a, b), while schizonts found in horses were smaller or the same size as the red blood cells (c). The *P. vivax* gametocyte was larger than the red blood cells found in buffalo (d). *P. falciparum* gametocyte and trophozoites (ring-shaped) with one or two nuclei was found in goats (e) and *P. falciparum* trophozoite found in horses had one nucleus (f).

## Discussion

*Plasmodium* presence was suspected in domestic animals because malaria cases in both Gaura and Fakfak villages remained high despite applying maximum preventive efforts including insecticide-treated bed nets. The results showed that 32 of the 270 blood (11.9%) samples contained human *Plasmodium*, serving as the first data report regarding this parasite, hence further investigation should be conducted.

Previous studies found *P. relictum* in avian species ( Cox, 2010), *P. cephalophi* in ungulates ( Bruce et al., 1913), *P. traguli* in mousedeer ( Garnham and Edeson, 1962), *P. brucei* in gray duiker ( Bruce et al., 1915; Templeton et al., 2016a), *P. bubalis* in water buffalo ( Sheather, 1919), and *P. odocoilei* in white-tailed deer ( Garnham and Kuttler, 1980; Perkins and Schaer, 2016). Other parasites detected included *P. caprae* in goats (ruminant) ( Kaewthamasorn et al., 2018), *P. bergei in* Rodentia ( Vincke and Lips, 1948), as well as *P. cynomolgi*, *P. inui*, and *P. fragile* in primates ( Dixit et al., 2018). The five *Plasmodium* species infecting humans were originally parasites in primates ( Duval et al., 2010; Knowlesi, 1935; Liu et al., 2014; Ng et al., 2008; Singh and Daneshvar, 2013; Zhang et al., 2016). This study identified *P. falciparum* in buffalos, goats, dogs, and horses, as well as *P. vivax* in buffalos, goats, and dogs. Initially, *Plasmodium* presence in the erythrocytes (RBCs) of these animals was uncertain, but the nested PCR produced the same results for all positive samples. Sequencing analysis of positive bands in the nested PCR confirmed the presence of *P. falciparum* and *P. vivax* ( Figure 3). The number of positive *Plasmodium* cases in buffalos was higher because of susceptibility to parasitic infections. This was consistent with the report of 10 parasitic infections observed by Mursyid in Central Lombok ( Mursyid et al., 2020). Another source declared age as one of the factors predisposing buffalos to high risk of infections ( Nurhidayah et al., 2019), with the average age exceeding seven years. This study provides the first report of human *Plasmodium* in ruminant, ungulate, and carnivorous domestic animals.

Investigations conducted by Templeton in Thailand in 2008 and 2015 showed the presence of *P. bubalis* among buffalos. The microscopic appearance of *P. bubalis* was depicted in the journal published by Templeton et al. (2016b). However, this current study did not detect any similarity between *P. bubalis* and *P. falciparum* gametocytes in buffalos. A molecular examination method targeting the cytochrome b (cytb) gene identified the presence of *P. caprae* in goats from Thailand, Myanmar, Iran, Kenya, and Sudan. The trophozoites of *P. caprae* can be observed in a journal published by Kaewthamasorn et al. (2018).

The discovery of *Plasmodium* among domestic animals in malaria-endemic areas raises the following questions. How do *P. falciparum* and *P. vivax* survive in these animals? Can animals serve as intermediate hosts for these parasites? Have both species evolved to live in ruminants, ungulates, and carnivores? Due to repeated exposure, have these animals become more susceptible to *Plasmodium*, which generally infects humans? Is *Plasmodium* pathogenic in animals?

*P. knowlesi* is identified as a commensal microbe in primates but pathogenic in humans ( Jongwutiwes et al., 2004; Ng et al., 2008; Singh and Daneshvar, 2013), and the transmission to humans can be attributed to forest loss or the invasion of primate habitats ( Davidson et al., 2019). There is a possibility that the proximity of animals and humans facilitates easier transfer of parasites between both groups through mosquitoes. In humans, *P. falciparum* and *P. vivax* infect by initially growing in liver cells before moving to RBCs. The parasites multiply in RBCs, leading to medical conditions characterized by fever, chills, headache, profuse sweating, weakness, rheumatic pain, symptoms of anemia or lack of blood, and nausea or vomiting. *Plasmodium* found in non-primates remains undetermined as pathogenic or commensal. However, sporozoites of *P. brasilianum* identified in animals migrate directly to the liver where multiplication occurs, releasing merozoites. The merozoites infect RBCs, initiating symptomatic disease in these animals ( Erkenswick et al., 2017). Organisms infected by *Plasmodium* become symptomatic when the parasite cycle advances to the erythrocyte stage or causes malaria due to the rupture of RBCs. The identification of intermediate hosts for *P. falciparum* and *P. vivax* in livestock is still a challenge. Furthermore, various studies attempted to provide evidence for the origination of these two species from chimpanzees and gorillas.

The investigation conducted by Prugnolle in 2013 signified that *P. vivax* detected in monkeys and humans had similarities. The results showed the possibility of natural transfer between the two organisms, particularly in environments where animals and humans coexist, facilitating continuous parasite transmission through vectors ( Prugnolle et al., 2013). Similarly, a study by Mu in 2005 described *P. vivax* as a zoonotic parasite ( Mu et al., 2005).

High API was observed in Fakfak and Gaura villages, but only animals from Gaura had human *Plasmodium.* This difference may be attributed to the long distance of approximately 50–500 m between the residential houses and animal enclosures in Fakfak. Meanwhile, in Gaura, residents live in stilt houses, with the ground floor and surroundings serving as a shelter for animals, which facilitate microbial transfer between humans and animals through mosquitoes. The inaccessibility of sampling locations and steep geographical conditions in Fakfak posed challenges during sample collections. Moreover, the vector census conducted in both areas identified 11 Anopheles mosquito species in Gaura village, including *An. vagus*, *An. sundaicus*, *An. aconitus*, *An. Kochi*, *An. flavirostris*, *An. indefinitus*, *An. maculatus*, *An. minimum*, *An. annularis*, *An. nivipes*, and *An. subpictus.* Molecular examination results showed that two *An. sundaicus* were infected by *Plasmodium*, while no *Anopheles* was found in Fakfak *.* The species *An. sundaicus* was identified as zoophilic in India ( Vidhya et al., 2019) and anthropophilic in Mekong, Vietnam ( Trung et al., 2005). These different reports from both areas suggested variations in the behavior of *An. sundaicus,* showing the diversity of mosquito biting patterns influenced by environmental factors. The proximity between domestic animals and humans in Gaura village may increase the tendency of bites from *An. sundaicus,* thereby necessitating further investigations.

The application of livestock for zooprophylaxis in malaria-endemic areas offers several advantages but tends to increase the survival of mosquitoes, which become potential disease vectors. Proximity between infected and uninfected organisms is a significant factor driving parasite transmission ( Kuris et al., 1980). The study by Hasyim suggests that livestock in endemic areas have a high potential to increase malaria incidence in Indonesia ( Hasyim et al., 2018). Moreover, zoopotentiation often occurs when livestock are kept indoors or near houses ( Donnelly et al., 2015). Locating livestock away from humans tends to reduce malaria cases ( Franco et al., 2014). Strategies for separating livestock from humans to minimize mosquito bites in both groups are essential. Mosquitoes that have fed on the blood of animals tend to not suck blood from humans. However, closeness between animals and humans can lead to massive pathogen transmission through intermediary vectors. Addressing factors such as vector abundance, mosquito survival, mosquito behavior, and local environment is crucial in mitigating zoonotic potential.

*Plasmodium* can be detected microscopically due to the smaller size of RBCs in animals compared to humans, but the molecular method is more significant for identifying the presence of this parasite. Nested PCR which offered high sensitivity similar to Real-Time PCR at a low cost was applied for the detection process ( Green and Sambrook, 2019; Perandin et al., 2004). The microscopic method using double fluorescent dyes with Giemsa stain is recommended for subsequent studies ( Guy et al., 2007).

Further investigation is needed to confirm the presence of domestic animal *Plasmodium* by amplifying the cytochrome B sequence and sequencing the invasion ligands such as DBL protein. Additionally, other challenges in malaria elimination shown by the results of this study should be addressed.

## Conclusion

In conclusion, *Plasmodium* species previously recognized as human parasites were detected among domestic animals in this study. The obtained results provided insights into malaria elimination challenges in the explored areas and were expected to promote public health improvement while guiding the development of malaria prevention strategies.

## Data Availability

Figshare: Underlying data for ‘The discovery of human *Plasmodium* among domestic animals in West Sumba and Fakfak, Indonesia’, https://doi.org/10.6084/m9.figshare.14703012.v3 ( Munirah et al., 2021). This project contains the following underlying data:
• Gel photo: Result of rPF1–RPF2 primers • Gel photo: Result of RPV1–rPV2 primers • Gel photo: Nested PCR *P. falciparum* and *P. vivax* *Figshare*: ARRIVE checklist for ‘The discovery of human *Plasmodium* among domestic animals in West Sumba and Fakfak, Indonesia’, https://doi.org/10.6084/m9.figshare.14703012.v3 ( Munirah et al., 2021). Data are available under the terms of the Creative Commons Attribution 4.0 International license (CC-BY 4.0).
