# Supplementary material for: Preliminary Evidence of Human Plasmodium in Domestic Animals from a Malaria-Endemic Region in Indonesia
Source: F1000Res. 2024 Dec 10;10:645. Originally published 2021 Jul 23. [Version 4] doi: 10.12688/f1000research.53946.4 (PMC11809677; doi:10.12688/f1000research.53946.4)
Supplement: Peer review history

# Reviewer report: Reviewer response for version 4

**DOI:** 10.5256/f1000research.175481.r351295

**Published:** 2025-01-09

**Authors:** Didik Sumanto

**Affiliations:** 1 Universitas Muhammadiyah Semarang, Semarang, Central Java, Indonesia

1. About claim: "the first data report". Comment: This claim seems unfounded because there have been previous findings in Etawa crossbred goats in Purworejo and cattle in Jayapura. Please check the following refer 1 and refer 3

 2. Figure 5: The microscopic image shown is not clear enough. The image cannot prove the morphological characteristics written in real terms. It is recommended that the re-shoot be done with a higher resolution. It would be better if each image of Plasmodium findings was accompanied by its microscopic ruler.

 3. It is recommended to create a table showing the results of microscopic tests and nested PCR tests for 32 positive samples to support this statement.

 4. The authors place more value on the genetic similarity between humans and animals in arguing to strengthen the finding of h-Plasmodium in domestic livestock. This is not wrong but the presence of zoophilic Anopheles plays an important role in transmitting h-Plasmodium from humans to animals. There needs to be a more explicit statement about the importance of zoophilic Anopheles' role as a cause of h-Plasmodium transmission in domestic livestock. Please see the following refer 2

Is the work clearly and accurately presented and does it cite the current literature?

Yes

If applicable, is the statistical analysis and its interpretation appropriate?

Yes

Are all the source data underlying the results available to ensure full reproducibility?

Yes

Is the study design appropriate and is the work technically sound?

Yes

Are the conclusions drawn adequately supported by the results?

Yes

Are sufficient details of methods and analysis provided to allow replication by others?

Yes

Reviewer Expertise:

Epidemiology, Parasitology, Malaria disease, Helminthiasis

I confirm that I have read this submission and believe that I have an appropriate level of expertise to confirm that it is of an acceptable scientific standard.

## References

[1] : Human-Plasmodium Like in Domestic-goat Blood in Malaria Endemic Areas in Purworejo Indonesia. *Journal of Communicable Diseases* .2021;53(04) : 10.24321/0019.5138.202185 148-152 10.24321/0019.5138.202185

[2] : Penularan Penyakit Malaria Oleh Vektor Zoofilik Dengan Sumber Pakan Darah Non Manusia. *PROSIDING SEMINAR KESEHATAN MASYARAKAT* .2023;1(Oktober) : 10.26714/pskm.v1iOktober.237 35-43 10.26714/pskm.v1iOktober.237

[3] : Utilisation and Delivery of Healthcare Services as Factors in Malaria Control in Kolar: A Community-Based Study. *Journal of Communicable Diseases* .2023;55(03) : 10.24321/0019.5138.202329 1-6 10.24321/0019.5138.202329

# Reviewer report: Reviewer response for version 3

**DOI:** 10.5256/f1000research.164578.r337062

**Published:** 2024-11-22

**Authors:** Paul Cliff Simon Divis

**Affiliations:** 1 Malaria Research Centre, Faculty of Medicine and Health Sciences, University of Malaysia, Sarawak, Malaysia

**General Comments**

 The manuscript explores the intriguing possibility of domestic animals serving as reservoir hosts for human Plasmodium species. While the research question is thought-provoking, significant issues in the rationale, methodology, and interpretation of results undermine the conclusions. The manuscript would benefit from a stronger theoretical framework that considers parasite-host coevolution and the evolutionary barriers to host switching.

**Specific Comments**

 In the introduction, the authors discuss the evolutionary origins of human Plasmodium species (P. falciparum, P. vivax, P. malariae, and P. ovale), tracing them to non-human primates, but this discussion is not effectively connected to the hypothesis that domestic animals could serve as reservoir hosts. Including a discussion of parasite-host coevolution would strengthen the argument. This discussion could highlight how human Plasmodium species are highly adapted to their primate hosts and emphasize the evolutionary and ecological barriers that make host-switching from primates to non-primate domestic animals unlikely. Addressing these barriers could challenge the hypothesis that primate Plasmodium species can adapt and persist in non-primate hosts.

 The rationale for hypothesizing that domestic animals might harbor human Plasmodium species needs to be more robust. Empirical or theoretical evidence should support why such a host switch might occur and whether this is biologically plausible.

 The qPCR methodology used in this study is inadequately described. It is important to clarify whether SYBR Green dye or probe-based assays were employed, as these methods differ in sensitivity and specificity. Details about primer design and validation should also be provided. When the primers were initially designed, they were not tested on Apicomplexan parasites from non-primate hosts, leaving their potential cross-reactivity with other parasites unknown. This is a critical limitation that should be addressed to ensure the validity of the findings.

 The authors should consider whether observed DNA signals might result from contamination, cross-reactivity, or amplification of related but non-Plasmodium DNA. Parasite-host coevolution poses significant challenges to the hypothesis that primate-adapted Plasmodium species can easily switch to non-primate hosts. The evolutionary pressures and physiological barriers that would make such a host switch unlikely should be discussed.

 The conclusion that domestic animals harbor human Plasmodium species is premature and unsupported by the data. A more robust genetic approach, such as sequencing longer DNA fragments across multiple genes, is necessary to confirm parasite identity. Short sequences are prone to misidentification, and the limitations of the current data should be acknowledged.

 Microscopy findings should be reported cautiously. It is inappropriate to label observed parasites as P. falciparum or P. vivax without further genetic confirmation. More neutral language, such as “resembling Plasmodium species,” is recommended. The title of the manuscript may also be misleading, as it suggests that human Plasmodium species have been conclusively identified in domestic animals. The title should reflect the preliminary nature of these findings.

 The reference to Mursyid et al. (2020) is inconsistent with the claims made in this study. Mursyid et al. did not report Plasmodium species infections in buffaloes, and this reference should be clarified or omitted.

 In my opinion, the authors should introduce parasite-host coevolution as a framework for evaluating the plausibility of host-switching events. This discussion could challenge the hypothesis and strengthen the manuscript’s theoretical basis. A more detailed and transparent description of the qPCR methodology is necessary, including primer validation to ensure specificity, especially given the lack of prior testing on Apicomplexan parasites from non-primate hosts. Genetic analyses should be expanded by sequencing longer DNA fragments from multiple genes to confirm species identity.

 The manuscript title and conclusions should better reflect the preliminary nature of the findings. Proof-reading is also needed to improve clarity and ensure that terminology and interpretations align with the presented data.

Is the work clearly and accurately presented and does it cite the current literature?

Partly

If applicable, is the statistical analysis and its interpretation appropriate?

Not applicable

Are all the source data underlying the results available to ensure full reproducibility?

Partly

Is the study design appropriate and is the work technically sound?

Partly

Are the conclusions drawn adequately supported by the results?

Partly

Are sufficient details of methods and analysis provided to allow replication by others?

Partly

Reviewer Expertise:

molecular epidemiology, population genetics, genomics, zoonotic malaria

I confirm that I have read this submission and believe that I have an appropriate level of expertise to confirm that it is of an acceptable scientific standard, however I have significant reservations, as outlined above.

# Response

**Published:** 2024-11-30

**Authors:** Sitti Wahyuni

**Affiliations:** Hasanuddin University, Indonesia

We sincerely appreciate your insightful feedback regarding the need for stronger empirical support for our hypothesis. In response to your suggestion, we have revised the section discussing host-switching and included additional evidence that explores similar host shifts observed in other parasite species, as well as the potential evolutionary constraints that may apply to Plasmodium species. Furthermore, we have incorporated recent studies on parasite-host coevolution, which we believe will provide greater depth to our argument.

 We also wish to thank you for your careful attention to the details of the qPCR methodology. To clarify, in this study, we utilized a SYBR Green-based qPCR approach, which is widely recognized for its sensitivity and cost-effectiveness in detection. We have now provided a more detailed description of the methodology in the revised manuscript, including the specific validation steps we undertook to ensure the accuracy of our results.

 As per your request, we have also revised the title and conclusion of the manuscript. While the primers were initially tested using Apicomplexan parasites from primate hosts, subsequent BLAST analysis suggested that the target sequences were highly conserved across various host species. Additionally, we employed the rPF1-rPF2 primers for P. falciparum and rPV1-rPV2 primers for P. vivax to amplify DNA fragments of 918 bp and 714 bp, respectively. These larger amplicons offer additional confidence in the assay's specificity. The results obtained from these primers can be accessed via the following link: https://doi.org/10.6084/m9.figshare.14703012.v3.

 While we were unable to perform sequencing of the PCR products with the rPF1-rPF2 and rPV1-rPV2 primers due to budgetary constraints, the PCR bands observed were consistent with the expected base pair lengths for P. falciparum and P. vivax.

 We greatly appreciate your constructive comments and believe these revisions have strengthened the manuscript.

# Reviewer report: Reviewer response for version 3

**DOI:** 10.5256/f1000research.164578.r264595

**Published:** 2024-05-14

**Authors:** Jaishree Raman

**Affiliations:** 1 National Institute for Communicable Diseases, University of the Witwatersrand Johannesburg, Johannesburg, Gauteng, South Africa; 2 UP Institute for Sustainable Malaria Control, WRIM,, University of Pretoria, Pretoria, Gauteng, South Africa

I have reviewed the revised article and think the authors have adequately addressed my concerns so am happy if this article is accepted.

Is the work clearly and accurately presented and does it cite the current literature?

Partly

If applicable, is the statistical analysis and its interpretation appropriate?

Not applicable

Are all the source data underlying the results available to ensure full reproducibility?

Partly

Is the study design appropriate and is the work technically sound?

Partly

Are the conclusions drawn adequately supported by the results?

Partly

Are sufficient details of methods and analysis provided to allow replication by others?

Yes

Reviewer Expertise:

Antimalarial drug and diagnostic resistance, molecular biology, molecular epidemiology, malaria elimination and control

I confirm that I have read this submission and believe that I have an appropriate level of expertise to confirm that it is of an acceptable scientific standard.

# Response

**Published:** 2024-05-18

**Authors:** Sitti Wahyuni

**Affiliations:** Hasanuddin University, Indonesia

Thank you for taking the time to review our revised article. We appreciate your constructive feedback and are pleased that we were able to address your concerns to your satisfaction. We look forward to the acceptance of our article.

# Reviewer report: Reviewer response for version 2

**DOI:** 10.5256/f1000research.144004.r163587

**Published:** 2023-11-28

**Authors:** Jaishree Raman

**Affiliations:** 1 National Institute for Communicable Diseases, University of the Witwatersrand Johannesburg, Johannesburg, Gauteng, South Africa; 2 UP Institute for Sustainable Malaria Control, WRIM,, University of Pretoria, Pretoria, Gauteng, South Africa

I thank the authors for revising the article based on the comments of two reviewers. While the scientific content of the manuscript has improved through the review process, it still requires correction in terms of English scientific writing. I recommend the authors enlist the assistance of an English editor to revise the article so that it meets the publication standards of the journal.

Is the work clearly and accurately presented and does it cite the current literature?

Partly

If applicable, is the statistical analysis and its interpretation appropriate?

Not applicable

Are all the source data underlying the results available to ensure full reproducibility?

Partly

Is the study design appropriate and is the work technically sound?

Partly

Are the conclusions drawn adequately supported by the results?

Partly

Are sufficient details of methods and analysis provided to allow replication by others?

Yes

Reviewer Expertise:

Antimalarial drug and diagnostic resistance, molecular biology, molecular epidemiology, malaria elimination and control

I confirm that I have read this submission and believe that I have an appropriate level of expertise to confirm that it is of an acceptable scientific standard, however I have significant reservations, as outlined above.

# Response

**Published:** 2024-04-03

**Authors:** Sitti Wahyuni

**Affiliations:** Hasanuddin University, Indonesia

Thank you for the feedback.

 We have submitted the manuscript to an English scientific writing service provider for language refinement. Subsequently, we have re-uploaded the revised file.

 Sincerely 

 Author

# Reviewer report: Reviewer response for version 1

**DOI:** 10.5256/f1000research.57382.r156443

**Published:** 2022-12-22

**Authors:** Jaishree Raman

**Affiliations:** 1 National Institute for Communicable Diseases, University of the Witwatersrand Johannesburg, Johannesburg, Gauteng, South Africa; 2 UP Institute for Sustainable Malaria Control, WRIM,, University of Pretoria, Pretoria, Gauteng, South Africa

This manuscript describes the interesting finding of human malaria parasites in domestic pets and farm animals in Indonesia. While this finding has potential implications for countries trying to stem residual transmission with the aim of being certified malaria free, this article needs some revision to ensure clarity and a logical flow of ideas. More information on the malaria burden and interventions in place in the study area need to be provided. Some possible reasons for the study findings need to be provided. The ideas for future studies should be presented as statements rather than questions.

 Below are some more specific comments:

 Introduction:

1. It is far too short. It needs to be expanded to explain the issue of residual malaria transmission in the study area and why it is concern. What interventions are in place to stop transmission and why you think they are failing to stem residual transmission.
2. There are four species of Plasmodia, not types that primarily affect humans
3. Is the statement on forest workers included because the study area has large numbers of forest workers? If so please provide this information.
4. From the data presented it looks like malaria transmission has decreased in both study areas so it is not clear to me what situation needs to be investigated. More clarity needs to be provided here.
5. A clear reason for the study needs to be provided as well as possible uses of the research findings.

Methods:

1. I am correct in assuming that the animal enclosures are in close proximity to the farmers homesteads?  Please give an average distance between enclosures and homes – as mosquitoes will only travel as far as they need to, to get their next blood meal.
2. The remaining is either blotted or spotted onto the filter paper. I do not understand what “the dry filter paper was put on a sterile paper clip” means.
3. Where the houses geo-located so you could map the infected animals?
4. How long was the incubation at 85 degrees?
5. It is DNA amplification by PCR not of PCR
6. For nest two is there no annealing stage?
7. Please explain why nest one products were run on a gel.
8. Where the qPCR products really analysed using gel electrophoresis?  The benefit of qPCR is real time data generation through the detection of fluorescence in the amplification stage.  The analysis of qPCR products is generally done by reading fluorescence outputs.
9. Please provide a bit more information on the rationale for the re-extraction of the sample. In addition please state if the additional blood spot was from the same blood spot or different blood spot?

Results

1. It is not clear whether the pigs were not infected will any form of malaria parasites or only not infected with human malaria parasites.  Please clarify.

Discussion

1. Please expand on the questions presented in the discussion in terms on possible results and their implication for malaria elimination in Indonesia and globally.
2. Please clarify if only infected were found in West Sumba region or only one study area within West Sumba.  The reason provided for only one area having infected animals is unclear to me.  Please rephrase for more clarity.
3. Please some guidance for future studies.

Is the work clearly and accurately presented and does it cite the current literature?

Partly

If applicable, is the statistical analysis and its interpretation appropriate?

Not applicable

Are all the source data underlying the results available to ensure full reproducibility?

Partly

Is the study design appropriate and is the work technically sound?

Partly

Are the conclusions drawn adequately supported by the results?

Partly

Are sufficient details of methods and analysis provided to allow replication by others?

Yes

Reviewer Expertise:

Antimalarial drug and diagnostic resistance, molecular biology, molecular epidemiology, malaria elimination and control

I confirm that I have read this submission and believe that I have an appropriate level of expertise to state that I do not consider it to be of an acceptable scientific standard, for reasons outlined above.

# Response

**Published:** 2023-02-06

**Authors:** Sitti Wahyuni

**Affiliations:** Hasanuddin University, Indonesia

*This manuscript describes the interesting finding of human malaria parasites in domestic pets and farm animals in Indonesia. While this finding has potential implications for countries trying to stem residual transmission with the aim of being certified malaria free, this article needs some revision to ensure clarity and a logical flow of ideas. More information on the malaria burden and interventions in place in the study area need to be provided. Some possible reasons for the study findings need to be provided. The ideas for future studies should be presented as statements rather than questions.*

*Below are some more specific comments:*

*Introduction:*

- *It is far too short. It needs to be expanded to explain the issue of residual malaria transmission in the study area and why it is concern. What interventions are in place to stop transmission and why you think they are failing to stem residual transmission.*

**Author’s response:** Thank you, we have made a revision.

  

- *There are four species of Plasmodia, not types that primarily affect humans*

 
**Author’s response:** Thank you for your input, we already revised. 

  

- *Is the statement on forest workers included because the study area has large numbers of forest workers? If so please provide this information.*

**Author’s response:** Thank you, we already revised.

  

- *From the data presented it looks like malaria transmission has decreased in both study areas so it is not clear to me what situation needs to be investigated. More clarity needs to be provided here.*

**Author’s response:** Thank you, we already revised.

  

- *A clear reason for the study needs to be provided as well as possible uses of the research findings.*

**Author’s response:** Thank you for your comment, we have revised accordingly.

*Methods:*

- *I am correct in assuming that the animal enclosures are in close proximity to the farmers homesteads?  Please give an average distance between enclosures and homes – as mosquitoes will only travel as far as they need to, to get their next blood meal.*

**Author’s response:** Thank you, we have already given the average distance in the method.

  

- *The remaining is either blotted or spotted onto the filter paper. I do not understand what “the dry filter paper was put on a sterile paper clip” means.*

**Author’s response: **Thank you, more clarity has been made.

  

- *Where the houses geo-located so you could map the infected animals?*

**Author’s response:** We apologize, because we don’t have the data about geo-location. But, in future research, we will apply this method. Thank you for your input.

  

- *How long was the incubation at 85 degrees?*

**Author’s response:** Thank you for your question, the incubation at 85 degrees was 10 minutes.

  

- *It is DNA amplification by PCR not of PCR*

**Author’s response:** Thank you for your comment, we have revised accordingly

  

- *For nest two is there no annealing stage?*

**Author’s response:** Thank you for your question, the annealing nested two was 55°C in one minute.


** **

- *Please explain why nest one products were run on a gel.*

**Author’s response:** Thank you. Actually, the nested one does not need to run on a gel, but it was done just to notice if there were any bands in the nested one. However, it is not the standard for determining whether positive at that stage, because we used nested two for determining *Plasmodium* positive.

  

- *Where the qPCR products really analysed using gel electrophoresis? The benefit of qPCR is real time data generation through the detection of fluorescence in the amplification stage.  The analysis of qPCR products is generally done by reading fluorescence outputs.*

**Author’s response:** Thank you for your question. Both products of nested and qPCR were analysed using gel electrophoresis and then run on agarose gel (nested one in 1.5%, nested two in 2% agarose gel and qPCR in 1.5% agarose gel) in order to identify fluorescence band wave in a gel documentation system.

  

- *Please provide a bit more information on the rationale for the re-extraction of the sample. In addition please state if the additional blood spot was from the same blood spot or different blood spot?*

**Author’s response:** Thank you, we already revised.

*Results*

- *It is not clear whether the pigs were not infected will any form of malaria parasites or only not infected with human malaria parasites.  Please clarify.*

**Author’s response:** Thank you, we already revised.

*Discussion*

- *Please expand on the questions presented in the discussion in terms on possible results and their implication for malaria elimination in Indonesia and globally.*

**Author’s response:** Thank you, we already revised.

  

- *Please clarify if only infected were found in West Sumba region or only one study area within West Sumba.  The reason provided for only one area having infected animals is unclear to me.  Please rephrase for more clarity.*

**Author’s response:** Thank you, we already revised.

  

- *Please some guidance for future studies.*

**Author’s response:** Thank you, we already revised.

# Reviewer report: Reviewer response for version 1

**DOI:** 10.5256/f1000research.57382.r101607

**Published:** 2022-01-06

**Authors:** Alfred Amambua-Ngwa

**Affiliations:** 1 Medical Research Council Unit, The Gambia at London School of Hygiene and Tropical Medicine, Fajara, The Gambia

The authors present data showing microscopically identified *Plasmodium* species in non-primate domestic animals in a region of Indonesia. They followed on to confirm infections with *Plasmodium* species with molecular methods targeting human malaria parasites *P. falciparum*, *P. vivax,* and *P. knowlesi*. They sequenced the amplified fragment, indicating that they indeed detected the genus. If this claim is validated, it will have significant consequences on malaria interventions and the goal of its elimination across the globe. The authors need to pay attention to the following to validate their claim:

1. Several *Plasmodium* species infect a wide range of animal species and there are other protozoa with similar cellular features that can also be microscopically misidentified for human *Plasmodium*. Thus, the identification of human *Plasmodium* will require species-specific distinguishing features by comparing the infected erythrocyte of the *Plasmodium*-infected domestic animals to those of other protozoan parasites including non-human *Plasmodium*.
2. A single sequence of each of the PCR products is shown per species and these sequences are similar to those of *Plasmodium falciparum* small rRNA. The alignment could include all samples sequenced and also how this was used to determine other species beyond *P. falciparum*. For speciation to be better validated, the authors could amplify and sequence cytochrome B, which is more divergent for *Plasmodium*.
3. More detail on the control protocols to limit contamination at sampling, extraction, and amplification of parasite DNA from the samples will help.
4. Given that humans and the family of domestic animals listed are largely divergent on RBC surface receptors for parasites, how these putative human-specific parasites invade the non-human hosts and develop will be of interest. The authors could therefore further validate their claim by sequencing an invasion ligand, such as the DBL proteins: EBA 175 for *P. falciparum* and DBA for *P. vivax*.
5. While the proximity between humans and animals is thought to be an explanation for the potentially zoonotic transmission, this will be surprising as the sharing of houses between humans and their domestic animals is a common occurrence in malaria-endemic tropical regions, where such a zoonosis has not been previously described. A census of the vector species between the two sites could enlighten on whether this potential zoonosis is driven by the vector.

Is the work clearly and accurately presented and does it cite the current literature?

Partly

If applicable, is the statistical analysis and its interpretation appropriate?

Not applicable

Are all the source data underlying the results available to ensure full reproducibility?

No

Is the study design appropriate and is the work technically sound?

Partly

Are the conclusions drawn adequately supported by the results?

Partly

Are sufficient details of methods and analysis provided to allow replication by others?

Partly

Reviewer Expertise:

Malaria, genomics, genetics, immunology, epidemiology, invasion, immunology

I confirm that I have read this submission and believe that I have an appropriate level of expertise to state that I do not consider it to be of an acceptable scientific standard, for reasons outlined above.

# Response

**Published:** 2023-02-06

**Authors:** Sitti Wahyuni

**Affiliations:** Hasanuddin University, Indonesia

Thank you for the suggestions and corrections. The corrections and suggestions were compiled and a revised version of the document is written. We believe that the method used has followed the malaria detection standards. The used methods have provided sufficient positive evidence so far. As for suggestions for validating our findings in several ways such as amplifying and sequencing cytochrome B, and sequencing an invasion ligand (such as the DBL proteins: EBA 175 for *P. falciparum* and DBA for *P. vivax*), we have included all in the discussion for further research because the method requires research funds which, for us, is quite a lot. Therefore, we are open to collaborate with other researchers from anywhere to work with us. We really appreciate the time spent by the reviewer to provide helpful comments and corrections.
